# Comparative genomics of *Synechococcus* and proposal of the new genus *Parasynechococcus*

**DOI:** 10.7717/peerj.1522

**Published:** 2016-01-14

**Authors:** Felipe Coutinho, Diogo Antonio Tschoeke, Fabiano Thompson, Cristiane Thompson

**Affiliations:** 1 Instituto de Biologia (IB), Universidade Federal do Rio de Janeiro (UFRJ), Rio de Janeiro, Brazil; 2 Centre for Molecular and Biomolecular Informatics (CMBI), Radboud Institute for Molecular Life Sciences, Nijmegen, The Netherlands; 3 COPPE/SAGE, Universidade Federal do Rio de Janeiro (UFRJ), Rio de Janeiro, Brazil

**Keywords:** Synechococcus, Phylogenomics, Genomics, Cyanobacteria, Genomic taxonomy

## Abstract

*Synechococcus* is among the most important contributors to global primary productivity. The genomes of several strains of this taxon have been previously sequenced in an effort to understand the physiology and ecology of these highly diverse microorganisms. Here we present a comparative study of *Synechococcus* genomes. For that end, we developed GenTaxo, a program written in Perl to perform genomic taxonomy based on average nucleotide identity, average amino acid identity and dinucleotide signatures, which revealed that the analyzed strains are drastically distinct regarding their genomic content. Phylogenomic reconstruction indicated a division of *Synechococcus* in two clades (i.e. Synechococcus and the new genus Parasynechococcus), corroborating evidences that this is in fact a polyphyletic group. By clustering protein encoding genes into homologue groups we were able to trace the Pangenome and core genome of both marine and freshwater *Synechococcus* and determine the genotypic traits that differentiate these lineages.

## Introduction

*Cyanobacteria* are unique among prokaryotes due to their ability to perform oxygenic photosynthesis. Members of this phylum are important contributors of global primary production, since they are responsible for a significant fraction of carbon fixation at aquatic habitats (Partensky, Hess & Vaulot, 1999; Richardson & Jackson, 2007). Among the members of this phylum, the sister genera *Synechococcus* and *Prochlorococcus* are often the most abundant members of the picophytoplankton (reaching concentrations up to 10^5^ and 10^6^ cells ml^−1^ respectively), being considered the most important contributors to CO_2_ fixation taking place in several oceanic regions (Li et al., 1983; Liu et al., 1998; Partensky, Blanchot & Vaulot, 1999). *Synechococcus* represents a polyphyletic group that encompasses both freshwater, seawater and brackish water lineages. The use of molecular data revealed that marine and brackish water *Synechococcus* strains are a sister clade to *Prochlorococcus*, that is distantly related to freshwater *Synechococcus* strains. Nevertheless, all these organisms are still classified under the same name (Honda, Yokota & Sugiyama, 1999; Robertson, Tezuka & Watanabe, 2001; Shih et al., 2013). Much of the comparative studies regarding *Synechococcus* strains were focused on marine lineages, and freshwater strains remained poorly characterized. Despite being sister taxa, *Prochlorococcus* and marine *Synechococcus*, differ regarding their ecology and biogeographical distribution patterns. Although these organisms frequently co-occur in aquatic environments, *Prochlorococcus* tends to be more abundant in warm and oligotrophic waters (Partensky, Blanchot & Vaulot, 1999; Bouman et al., 2006). Marine *Synechococcus* is considered to be ubiquitously distributed, due to its presence in estuarine, coastal and off-shore waters, broader temperature range and high abundance at mesotrophic and eutrophic habitats (Six et al., 2007a).

Genomic studies encompassing both the marine and the distantly related freshwater strains, further expanded the knowledge on the genetic diversity within *Synechococcus*. Such studies revealed that these organisms developed unique strategies to adapt to their respective environments, that involve several aspects of their metabolism and physiology, e.g. uptake and utilization of nutrients and metals, regulatory systems and motility (Palenik et al., 2006; Six et al., 2007a; Dufresne et al., 2008a). These strains can also be differentiated with regard to their ecology. Several studies have demonstrated differential patterns of biogeographical and seasonal distribution among *Synechococcus* strains, that are believed to be driven by environmental conditions such as depth, salinity, temperature and nutrient availability (Tai & Palenik, 2009; Paerl et al., 2011; Post et al., 2011).

One of the factors that may have contributed to the remarkable diversity of both freshwater and marine *Synechococcus* is horizontal gene transfer (HGT). It has been demonstrated that this process plays a significant role into the evolution of *Cyanobacterial* genomes (Nakamura et al., 2004; Zhaxybayeva et al., 2006). Furthermore, genomic islands have been identified in several genomes of *Cyanobacteria*, that are thought to have been acquired during infection by Cyanophages, as evidenced by the presence of integrases flanking these regions (Palenik et al., 2003; Palenik et al., 2009). Genes carried by phages, have the potential to be horizontally transferred and may be associated with several metabolic processes, e.g. photosynthesis, carbon and phosphorus metabolism and stress response (Mann et al., 2003; Lindell et al., 2004; Sullivan et al., 2005; Palenik et al., 2003; Dufresne et al., 2008a).

Comparative genomics has successfully been applied to several groups of organisms, allowing for the identification of new species, reconstruction of phylogenies and definition of genomic traits that are responsible for the metabolic and ecological differences observed between these organisms (Chen et al., 2006; Makarova et al., 2006; Thompson et al., 2009; Thompson et al., 2013; Thompson et al., 2014). Bacterial taxonomy applies a polyphasic approach, i.e. integration of phenotypic, genotypic and phylogenetic data, for the classification of microorganisms, based on traits that range from the molecular to the ecological level (Colwell, 1970; Vandamme et al., 1996). Microbial taxonomy has come to incorporate whole genome information, giving rise the field of genomic taxonomy, that uses the massive amounts of information contained in complete genome sequences, for the classification and differentiation between microbial lineages (Coenye et al., 2005). Nevertheless, the very concept of bacterial species remains elusive, and therefore the classification of bacteria into species, based on genomic or any other type of feature, remains challenging.

A total of 24 complete *Synechococcus* genomes have been sequenced until the year of 2013. These sequences were obtained from strains isolated from several habitats throughout the globe, each possessing unique genetic, metabolic and ecologic traits. Despite all being named *Synechococcus*, these genomes represent a polyphyletic group and the genetic similarities, and differences between these strains have not been well characterized in a broad-scale comparative genomic analysis. By identifying groups of homologous genes shared between these genomes, we were able to trace the core-genome and the pan-genome of *Synechococcus* and *Prochlorococcus*. Based on these results and on phylogenomic reconstruction, we propose the creation of the genus *Parasynechococus*, a sister clade to *Prochlorococcus*.

## Materials and Methods

### Samples

A total of 24 complete *Synechococcus* genomes publicly available as of August 2013 were retrieved from Genbank for analysis (Table 1). Only two genomes were classified at the species level: *Synechococcus elongatus* strain PCC6301 and *Synechococcus elongatus* strain PCC7942. The genomes were sequenced from isolates obtained from several aquatic environments around the globe, including freshwater, coastal and open water marine environments and covering a depth range from 0 to 1,800 meters. These genomes show marked variation regarding their size (2.12–5.97 Mb), G+C content (48.20%–65.40%) and amount of protein encoding genes (2,510–5,702) (Table 1), suggesting that they may have originated from organisms with long divergence times.

**Table 1:** Characteristics of the 24 strains used for comparative genomics regarding: strain, source of isolation, NCBI accession identification, number of scaffolds, genome size, GC content and number of identified coding DNA sequences.

Organism/Strain	Source	NCBI Accession number	Scaffolds	Length (Mbp)	GC(%)	CDS
Synechococcus elongatus PCC 6301	Freshwater	NC_006576.1	1	2.7	55.50	2901
Synechococcus elongatus PCC 7942	Freshwater	NC_007604.1	2	2.74	55.46	2882
BL107	Blanes Bay, Mediterranean Sea, 1,800 m	NZ_DS022298.1	1	2.29	54.20	2655
CB0101	Chesapeake Bay	NZ_ADXL00000000.1	94	2.69	64.20	2881
CB0205	Chesapeake Bay	NZ_ADXM00000000.1	78	2.43	63.00	2661
CC9311	California current, Pacific (coastal), 95 m	NC_008319.1	1	2.61	52.40	3164
CC9605	California current, Pacific (oligotrophic), 51 m	NC_007516	1	2.51	59.20	3016
CC9902	California current, Pacific (oligotrophic), 5 m	NC_007513	1	2.23	54.20	2635
JA23Ba213	Octopus Spring, Yellowstone Park	NC_007776	1	3.05	58.50	3064
JA33Ab	Octopus Spring, Yellowstone Park	NC_007775.1	1	2.93	60.20	3036
PCC 6312	Freshwater, California	CP003558.1	2	3.72	48.49	3795
PCC 7002	Unknown	NC_010475.1	7	3.41	49.16	3008
PCC 7335	Snail shell, intertidal zone, Puerto Penasco, Mexico	ABRV00000000.1	11	5.97	48.20	5702
PCC 7336	Sea Water Tank, Berkey University	ALWC00000000.1	1	5.07	53.70	5093
PCC 7502	Sphagnum bog	CP003594.1	3	3.58	40.60	3703
RCC307	Mediterranean Sea, 15 m	NC_009482.1	1	2.22	60.80	2571
RS9916	Gulf of Aqaba, Red Sea, 10 m	NZ_DS022299.1	1	2.66	59.80	2927
RS9917	Gulf of Aqaba, Red Sea, 10 m	NZ_CH724158.1	1	2.58	64.40	2719
WH 5701	Long Island Sound, USA	NZ_CH724159–NZ_CH724167	116	3.28	65.40	3185
WH 7803	Sargasso Sea, 25 m	NC_009481	1	2.37	60.20	2660
WH 7805	Sargasso Sea	NZ_CH724168.1	3	2.63	57.60	2931
WH 8016	Woods Hole, MA, USA	AGIK00000000.1	16	2.69	54.10	3184
WH 8102	Sargasso Sea	NC_005070.1	1	2.43	59.40	2752
WH 8109	Sargasso Sea	ACNY00000000.1	1	2.12	60.10	2510

### Genomic taxonomy

The genomes of the 24 strains were compared through six methods: I) Average Nucleotide Identity (Konstantinidis & Tiedje, 2005a; Konstantinidis & Tiedje, 2005b), II) Average Amino acid Identity (Konstantinidis & Tiedje, 2005a) III) Dinucleotide signature (Karlin, Mrazek & Campbel, 1997), IV) *in silico* DNA-DNA hybridization (Meier-Kolthoff et al., 2013), V) Identity of *rrsA* gene sequences (16S rRNA gene) and VI) Multi-locus sequence analysis (MLSA) of genes *rrsA, gyrB, pyrH, recA* and *rpoB*. The Cgview comparison tool (Grant, Arantes & Stothard, 2012) was used to create a genome-wide homology map, based on protein identity using *S. elongatus* PCC 7942 as the reference genome. Synteny between *Prochlorococcus* and *Synechococcus* genomes was analyzed through whole-genome alignments performed in progressiveMauve (Darling, Mau & Perna, 2010).

To facilitate analyzes for genomic taxonomy we developed a program written in Perl named GenTaxo, freely available at sourceforge.net/projects/gentaxo/. This tool receives as input genome sequences in FASTA format to calculate three metrics for genome comparison: Average Nucleotide Identity (ANI), Average Amino acid Identity (AAI) and distances between genomes based on dinucleotide signature.

### Homologue identification

The 82,703 proteins encoded in 24 *Synechococcus* and 13 *Prochlorococcus* genomes (Thompson et al., 2013) were analyzed through OrthoMCL v1.4 (Li, Stoeckert & Roos, 2003), allowing for the identification of both orthologous and paralogous genes shared between these taxa. Homologue identification was also performed between *Synechococcus* OrthoMCL was run using the following parameters: inflation factor of 1.25 and e-value ≤10^−05^.

### Phylogenomic reconstruction

Orthologous groups identified by OrthoMCL were used to reconstruct the phylogeny of *Synechococcus* and *Prochlorococcus* genomes. Protein sequences of 607 orthologous genes shared between *Synechococcus* and *Prochlorococcus* (with no identified paralogs) were aligned through MUSCLE (Edgar, 2004). Next, protein alignments were converted to nucleotide alignments through pal2nal (Suyama, Torrents & Bork, 2006) and each of the 607 alignments were concatenated. Distances between taxa were calculated through the Tajima-Nei method using the concatenated alignment. The phylogenomic tree was reconstructed through the Neighbor-joining algorithm using MEGA5. Bootstrap tests were performed in 1,000 replicates.

## Results

### Genomic taxonomy

The results from the different methods of comparative genomic analysis all indicated the same pattern: the analyzed strains were extremely distinct regarding genomic content. With the exception of *Synechococcus elongatus* PCC6301 and *Synechococcus elongatus* PCC7942, the remaining pairs of strains presented values for ANI and AAI drastically below the species cutoff (95% for both methods). The results obtained from DDH and dinucleotide signatures corroborated these patterns as all the strains had an estimated level of DNA-DNA hybridization below 70% (expect for the aforementioned pair) and the majority of genome pairs yielded distances based on dinucleotide content above the 0.01 cutoff. The same trend was observed through MLSA and 16S rRNA gene comparisons.

### Phylogenomic reconstruction

**Figure 1:**
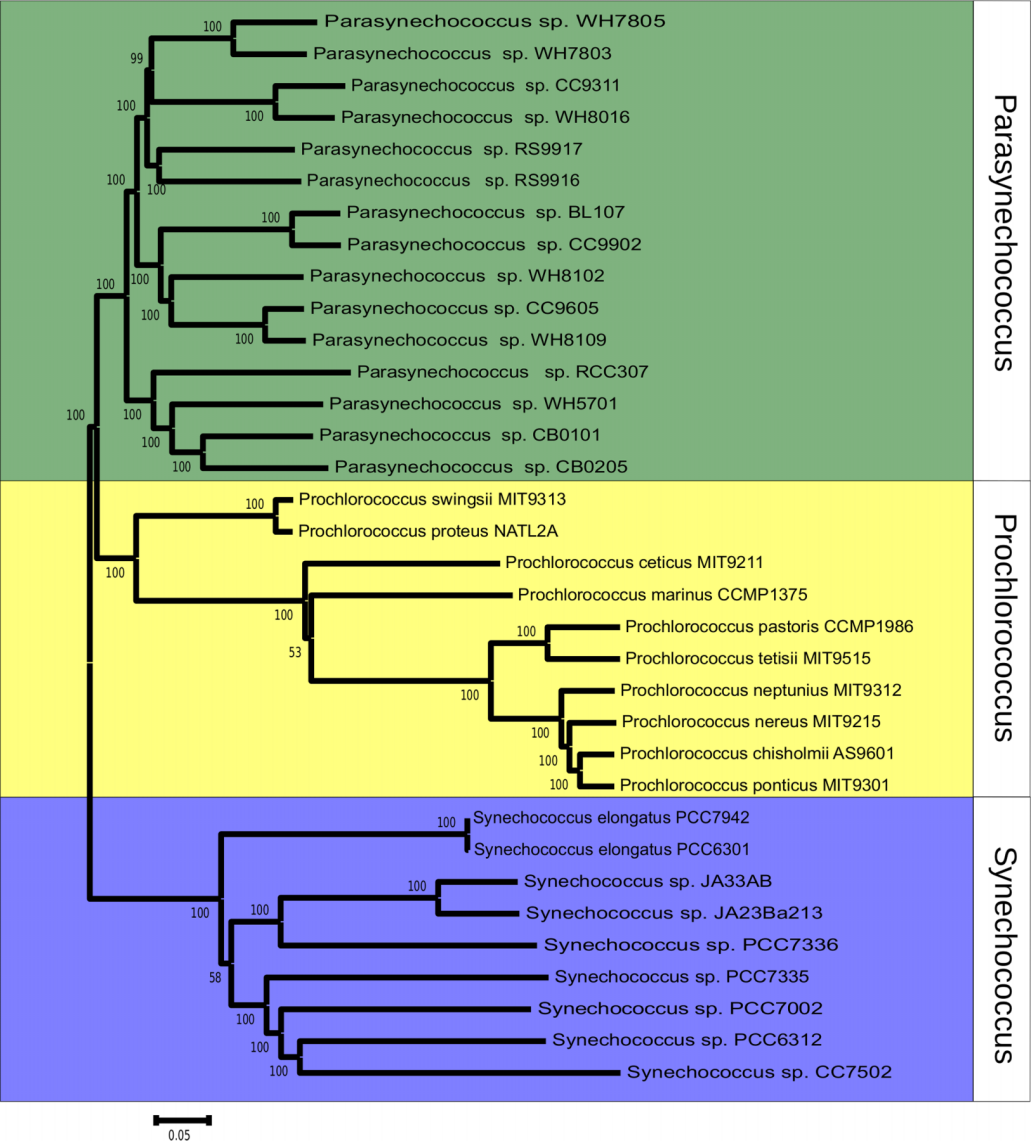
Phylogenomic reconstruction 37 *Synechococcus* and *Prochlorococcus* strains based on the concatenated alignment of 607 ortholog genes. The tree was constructed through neighbor-joining using the Tajima-Nei method. Bootstrap tests were conducted with 1000 replications.

The phylogeny of *Synechococcus* was reconstructed based on the sequences of 607 orthologous genes, with no paralogs, shared between *Synechococcus* and *Prochlorococcus* genomes (Fig. 1). Bootstrap values were above 70% for the majority of nodes, indicating very high consistency of topology. Two major clades of *Synechococcus* could be identified. The first, made up mostly of marine strains: CB0101, CB0205, WH5701, RCC307, RS9917, RS9916, WH7805, WH7803, BL107, CC9902, WH8102, CC9605, WH8109, WH8016 and CC9311. The second clade is made up mostly of freshwater strains: JA23Ba213, JA33AB, PCC7336, PCC7942, PCC6301, PCC7335, PCC7002, PCC7502 and PCC6312. Tree topology suggested that the 24 genomes represent a polyphyletic group that can be divided in marine strains, a sister taxa of *Prochlorococcus*, and freshwater and inter-tidal strains.

### Homologue identification

The 82,703 protein encoding genes analyzed represented the pan-genome of the *Synechococcus* and *Prochlorococcus*, the diversity of all protein encoding genes of these genomes. This analysis identified 8,167 homologous groups (Table S1), of those, 744 were shared between all lineages, thus representing the core-genome of *Synechococcus* and *Prochlorococcus*. Out of all genes, 15,724 are exclusive of a single genome, of which 577 have a paralog within the same genome and 15,147 are orphans, i.e. are exclusive of a single genome and have no identified orthologs or paralogs (Table S2).

### Genomic map and synteny

**Figure 2:**
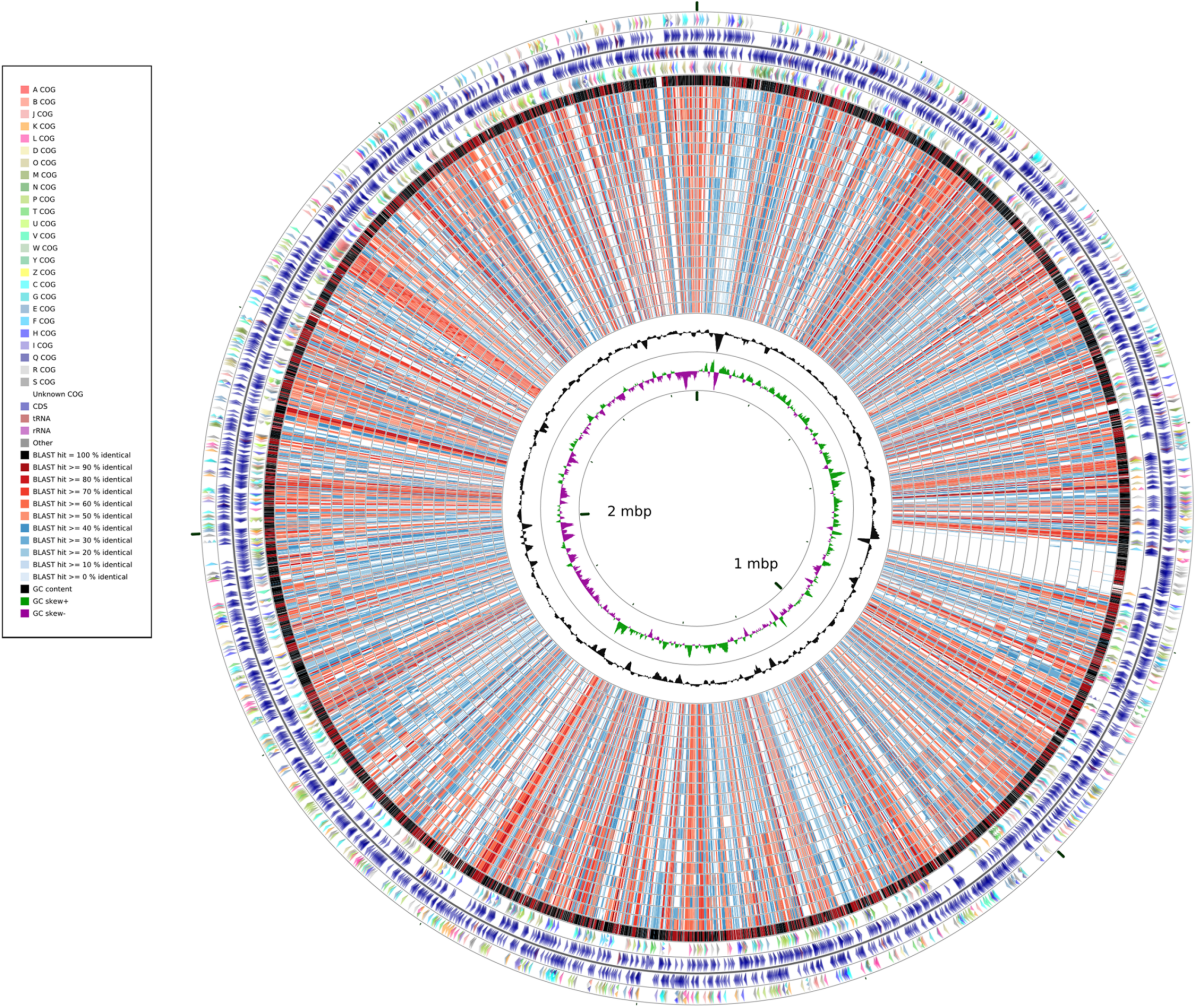
Whole-genome identity map generated through Cgview comparison tool. Protein sequences of each strain were compared against the genome o PCC7942 through blastx. Rings represent, from outermost to innermost: 1) Genes of the plus strand color coded by COG category; 2) Protein encoding and RNA genes at the plus strand; 3) Genes of the minus strand color coded by COG category; 4) Protein encoding and RNA genes at the minus strand; 5) PCC6301; 6) PCC7335; 7) PCC6312; 8) PCC7002; 9) PCC7336; 10) PCC7502; 11) WH5701; 12) JA23Ba213; 13) JA33Ab; 14) WH7805; 15) RS9917; 16) WH7803; 17) RCC307; 18) RS9916; 19) WH8016; 20) CC9605; 21) CC9311; 22) WH8102; 23) BL107; 24) WH8109; 25) CC9902; 26) G+C Content; 27) G+C Skew.

A whole-genome identity map was created by comparing all the genomes against that of *Synechococcus elongatus* PCC7942 through blastx (Altschul et al., 1990). This analysis (Fig. 2) revealed low identity levels (<80%) across the extension of the genomes, and indicated several sites of potential insertion/deletion events at the genome of *Synechococcus elongatus* PCC7942. Even though *S. elongatus* PCC6301 and *S. elongatus* PCC7942 are very closely related, the map illustrated that their genomes were not completely identical. While the majority of their genomes shows very high identity levels (>90%), as demonstrated by the dominance of black and dark red colors of the outermost circle, several segments appeared to be exclusive of PCC7942 and not present in PCC6301, which are examples of variation of genomic content between organisms that have very short divergence times.

Whole-genome alignments between marine and freshwater *Synechococcus* revealed little to no synteny between the genomes of these lineages and a significant amount of genome rearrangement events occurring between strains (Figs. S2A and S2B). This pattern was not observed when analyzing *Prochlorococcus* genomes, which showed a somewhat syntenic genome organization (Fig. S2C).

## Discussion

### Phylogenomics of Synechococcus and Prochlorococcus

The phylogenomic reconstruction based on the concatenated alignments of 607 orthologs separated the 24 Synechococcus genomes into two major groups, roughly segregating seawater and freshwater strains, and is supported by high bootstrap values (Fig. 1). This pattern points toward a division between these two lifestyles early on the evolutionary history of these organisms. Previous studies that investigated the evolution of *Synechococcus* suggested that marine strains are closely related to *Prochlorococcus* and distant from members of freshwater strains, which would make *Synechococcus* a polyphyletic group that encompasses at least two genera (Honda, Yokota & Sugiyama, 1999; Robertson, Tezuka & Watanabe, 2001; Fuller et al., 2003; Shih et al., 2013), that can be further divided into several subclades (Fuller et al., 2003; Shih et al., 2013; Matzke, Shih & Kerfeld, 2014). The phylogeny was reconstructed based on the genes of the core-genome of *Prochlorococcus* and of both marine and freshwater *Synechococcus* strains. These 607 ortholog groups encode constitutive functions and genes that are very unlikely to undergo HGT. Nevertheless, to rule out the possibility that the segregation between the two lineages emerges from environment specific HGT, a second tree was constructed, using concatenated alignments of *pyrH, recA* and *gyrB* genes (Fig. S1). This tree further corroborates the consistency of the obtained topology.

### Pan and core genome composition of Prochlorococcus and Synechococcus

Previous studies have investigated the pangenome of marine *Synechoccocus* and of *Cyanobacteria* as a whole. These analyzes estimated a core-genome of 1,228 orthologue groups for marine *Synechococcus* and *Prochlorococcus* (Dufresne et al., 2008b) and of 892 orthologue groups for *Cyanobacteria* (Mulkidjanian et al., 2006). Our results point to a comparable figure, i.e. a core genome for marine and freshwater *Synechococcus* of 866 orthologue groups that drops to 744 when *Prochlorococcus* is included. The somewhat smaller core-genome obtained here is a consequence of using a larger number of genomes that belong to a broader range of phylogenetic lineages (Shih et al., 2013). These studies also came to similar conclusions regarding the functional roles of the orthologue groups which make up the core-genome, many of which encode genes associated to essential physiologic functions, e.g. photosynthesis and DNA metabolism, cell division, circadian cycle and ribosomal proteins. Meanwhile the non core orthologue groups are usually involved in habitat adaptation and nutrient uptake (Mulkidjanian et al., 2006; Dufresne et al., 2008b).

### *Genetic distinctions between* marine *Synechococcus/Prochlorococcus* and freshwater *Synechococcus*

A total of 310 orthologue groups are present in all marine *Synechococcus* and *Prochlorococcus* but absent from at least one freshwater *Synechococcus* genome (Table S1). Among the traits that differentiate these two clades, their carbon concentration mechanisms are among the most relevant. *Cyanobacteria* have carboxysomes, intracellular microcompartments formed by a protein shell that encapsulates the enzymes carbonic anhydrase and ribulose-1,5-bisphosphate carboxylase/oxygenase (RuBisCO). Carbonic anhydrase converts ${\rm{HCO}}_{3^{-}}$ to CO_2_ around RuBisCO that in turn utilizes this CO_2_ molecule to synthesize phosphoglycerate. The compartmentalization brought by the carboxysome, enhances the efficiency of carbon fixation by elevating the levels of intracellular CO_2_ around RuBisCO (Rae et al., 2013). Two forms of carboxysome have been identified that differ regarding their enzymes, transporters, structural proteins and the RuBisCO isoform within them. Marine *Cyanobacteria* have α-Carboxysomes while coastal and freshwater species harbor β-Carboxysomes (Yeates et al., 2008). Genes encoding Carboxysome shell proteins CsoS2 and CsoS3 and Carboxysome peptides A and B are present in all the genomes marine *Synechococcus* and *Prochlorococcus*, these proteins are characteristic of α-Carboxysomes. Meanwhile proteins of the *ccm* operon (L, M, N and O), which are characteristic of β-Carboxysomes were found exclusively and in all of the members freshwater *Synechococcus* clade. These findings indicate that marine and freshwater *Synechococcus* use distinguished apparatus for carbon concentration, meaning that these groups could have differential contributions regarding their roles as primary producers and to the global biogeochemical carbon cycle. Although, despite the different protein composition of α and β carboxysomes, functional differences between them are still poorly characterized (Yeates et al., 2008).

A total of 46 orthologue groups are found exclusively on genomes of *Prochlorococcus* and marine *Synechococcus* clade and absent from all freshwater *Synechococcus* lineages. Among those, the ones with well characterized functions include proteins such as: a Fructokinase (carbohydrate metabolism), an inorganic pyrophosphatase (phosphorus metabolism), a carboxypeptidase (protein degradation and maturation), an aspartocyalase (aspartic acid biosynthesis), GMC oxidoreductase (carbohydrate metabolism), a Ribonuclease (tRNA maturation), and an RpoD like sigma factor (transcriptional regulation). A total of 71 orthologue groups are found exclusively and in all genomes of freshwater *Synechococcus,* the ones with well characterized functions include: a Lycopene cyclase (carotenoid biosynthesis), a RecJ exonuclease (DNA repair), an oligopeptide permease (peptide transport), a citramalate synthase (aminoacid biosynthesis), a folate transporter and several ABC transporters.

### Genetic distinctions between marine and freshwater Synechococcus

Besides carboxysome structure, marine Synechococcus can be differentiated from freshwater *Synechococcus* by: 16S rRNA gene homology to marine strains of *Synechococcus*, absence of Fructose-1,6-bisphosphatase and 6-phosphofructokinase and presence of DNA polymerase II (not detected in any of the other *Synechococcus* genomes) and also by the presence of 101 identified homologs groups that are specific to either one of these two lineages. Many of these genes are involved in essential metabolic processes, i.e. carbohydrate metabolism, nutrient uptake, transcriptional regulation and DNA replication. Nevertheless, molecular assays would be required to describe how the physiology of these organisms is affected by the presence/absence of these proteins, which unfortunately is outside of our scope. However it is reasonable to assume that these organisms make use of alternative proteins to fulfill those roles, which could belong to the many poorly characterized and hypothetical proteins that are present in these genomes (Tables S1 and S2).

Two-component response regulators are among the mechanisms used by bacteria to sense environmental stimulus and adapt accordingly by modulating protein activity through alteration of transcriptional patterns (West & Stock, 2001; Laub & Goulian, 2007). Freshwater *Synechococcus* and marine *Synechococcus* strains differ regarding the presence and abundance of proteins of two-component response regulators, histidine kinases and transcriptional regulators (e.g. sigma factors). These orthologue groups are indicated by stars on column 4 of Tables S1 and S2. Unfortunately, the specific roles of the majority of these proteins remains unknown. Nevertheless, the fact that each strain harbors a distinctive set of these regulators points to unique responses to environmental stimuli and mechanisms for transcriptional regulation for each of them.

### Phenotypic and ecological traits of marine and freshwater Synechococcus

An extensive body of knowledge has been focused on characterizing differences and similarities between *Synechococcus* strains based on phenotypical and ecological traits. Most of this work has been focused on marine and coastal strains, and unfortunately freshwater strains remain poorly characterized. Nevertheless, those phenotypic analyzes corroborate our results, as they all point to significant distinction between the *Synechococcus* lineages. A time-series study of the abundance patterns of the clades that include lineages CC9311, CC9605, WH8102 and CC9902 at a coastal Pacific ocean environment revealed that these organisms have distinctive temporal distribution patterns throughout the year, with the clades that include strains CC9311 and CC902 emerging as dominant members of the community while the clades of WH8102 and CC9605 appear as low-abundance members throughout most of the time series (Tai & Palenik, 2009). Another time series study performed at the Gulf of Aqaba also reported uneven distribution of *Synechococcus* clades throughout the year, and linked fluctuations in the abundance of these lineages to their preferences regarding nutrient utilization strategies (Post et al., 2011). A study focused on the spatial distribution of several clades of marine *Synechococcus* (which include some of the strains analyzed here, namely WH8016, CC9311, WH8109, RS9917 and WH8102) across oceanic provinces provided strong evidence for distinctive distribution signatures across the oceans for each one of the analyzed clades, which could be driven by differences in the capacities of these organisms to adapt to nutrient availability and temperature (Zwirglmaier et al., 2008).

Phycobilisomes are light-harvesting complexes present in Cyanobacteria. These structures are formed by a Phicocyanin core that can be linked phycobiliproteins and phicoerythrin. These structures are responsible for absorbing energy from light that is transferred to Chlorophyll molecules (Glazer, 1985; Mullineaux, 2008). Therefore, phycobilisome structure determines the light spectra that can be used by a given organism, and consequently its capacity to photosynthesize at different environments. Eleven marine *Synechococcus* strains have had their phycobilisome structures analyzed and compared, revealing that even within this group of closely related organisms there is a remarkable diversity regarding their light-absorption apparatus (Six et al., 2007b). The functioning and tolerance of fluctuations in irradiance of the light harvesting apparatus of lineages WH8102, RS9917 and RCC307 has been shown to be distinct between them and also different from that of *Prochlorococcus.* These differences are thought to be associated with niche-partitioning between these organisms, that make use of distinct light spectra for photosynthesis (Six et al., 2007a).

### Horizontal gene transfer influences the evolution of Synechococcus

Mobile genetic elements and horizontal gene transfer play a significant role at the evolution of *Cyanobacteria* (Zhaxybayeva et al., 2006; Palenik et al., 2009). Among the homologous groups identified, many of those that show drastic differences in their number of copies at each genome are associated with mobile genetic elements. As an example, an homologue group encoding for a transposase was detected exclusively in the genome of strain PCC 7335. Thirty-five copies of this gene were detected, making it the protein with most copies in this genome. Three other homologue groups encoding integrases were more abundant in the genome of PCC 7335 than any of the others analyzed genomes. Interestingly, PCC 7335 has the largest genome among the analyzed strains followed by PCC 7336, whose genome if also filled with multiple copies of transposases. This pattern suggests that these transposases may be responsible for the increased genome of these strains, as these elements mediate acquisition of exogenous DNA (Ochman, Lawrence & Groisman, 2000; Juhas et al., 2009). Besides these integrases, many homologous groups identified are also involved in gene transfer events (e.g. plasmid proteins, CRISPR, transposons and phage proteins). Altogether, these results provide evidence that horizontal gene transfer agents are important drivers of the evolution of these genomes, contributing to the diversification of the group, these elements may be one of the sources of the extensive genomic plasticity found among marine and freshwater *Synechococcus*.

Bacterial genomes are dynamic, constantly undergoing contraction through gene loss and expansion mediated by horizontal gene transfer (Puigbò et al., 2014). Recent studies have explored the drastic genome reduction that occurred during the evolution of *Prochlorococcus* (Kuo, Moran & Ochman, 2009; Batut et al., 2014). However, no evidence for such process has been observed for either clade of *Synechococcus*. Instead, our data points to an opposite trend among these taxa, acquisition of new genes leading to enlargement of genomes driven by invasion of exogenous DNA through horizontal gene transfer. This invasive DNA molecules are able to fixate in species with small effective population sizes, in which genetic drift is more relevant than natural selection for genome evolution (Batut et al., 2014). Considering the superior cell densities of *Prochlorococcocus* compared to *Synechococcus*, genetic drift is expected to be less influential over the first than the letter, thus favoring a reduced genome size as consequence of strong natural selection. These distinctions of genome size between *Synechococcus* and *Prochlorococcus* may be associated with the environmental distribution of these organisms. The first are typical of freshwater and coastal environments, that are richer in nutrients than the oligotrophic waters occupied by the latter. Therefore, the selective pressure towards a reduced genome may be more pronounced over *Prochlorococcus* since it thrives in nutrient deprived environments.

### A new taxonomic classification for Synechococcus

The results from ANI, AAI, and in silico DDH analyzes indicate that, with the exception of strains PCC7942 and PCC6301, the level of dissimilarity found between these genomes suggests very long diverge times. Such a trend is also corroborated by the 13,511 identified orthologous groups that can differentiate these lineages. It is therefore likely that these genomes represent different species, which according to the phylogenomic reconstruction can be segregated into two different genera: a sister clade to *Prochlorococcus* formed by the marine strains with the proposed name *Parasynechococcus*, and a second clade formed by freshwater *Synechococcus* strains.

A formal description of *Parasynechococcus* following the criteria established by the International Journal of Systematic and Evolutionary Microbiology follows:

1. List of the strains included in the taxon: Based on the phylogenomic reconstruction, the following strains are assigned to *Parasynechococcus*: CB0101, CB0205, WH5701, RCC307, RS9917, RS9916, WH7805, WH7803, BL107, CC9902, WH8102, CC9605, WH8109, WH8016 and CC9311.
2. Tabulation of the characteristics of each strain: Tables S1 and S2 list the collection of orthologous groups that can be used to differentiate between these strains based on their genomic content.
3. List of characteristics considered essential for membership in the taxon: Characteristic traits of *Parasynechococcus* are: 16S rRNA gene closely related to marine strains of *Synechococcus* and *Prochlorococcus*, α-Carboxysomes, absence of Fructose-1, 6-bisphosphatase and 6-phosphofructokinase and presence of DNA polymerase II. Also the orthologue groups described in Tables S1 and S2 can be used to differentiate this genera from *Prochlorococcus* and freshwater *Synechococcus* and also between the Parasynechococcus 15 strains.
4. List of characteristics which qualify the taxon for membership in the next higher taxon:
  Both *Prochlorococcus* and *Parasynechococcus* belong to the Synechococcaceae family, order *Chroococcales*. These organisms are grouped together on the basis of phylogenetic reconstruction, nevertheless the phenotypic and genotypic traits that distinguish this family remain poorly characterized.
5. List of diagnostic characteristics: Tables S1 and S2 also list the genomic traits that distinguish *Paraynechoccus* from the sister taxa *Prochlorococcus*.
6. Designation of the type for that taxon: Strain WH8102 was chosen as the type strain of Parasynechococcus. This strain represents the first complete genome of the genus to be sequenced, has reasonable amount of descriptive data encompassing several aspects of its biology, such as carboxysome structure (Iancu et al., 2007), the light-harvesting apparatus (Six et al., 2007b), seasonal abundance patterns (Tai & Palenik, 2009; Post et al., 2011), and nutrient uptake and utilization (Moore et al., 2005; Su et al., 2006; Tetu et al., 2009). Also, as required, this strain is available in two international culture collections (Roscoff Culturing Collection, France and NCMA, USA).
7. Reactions of the type strain: To our knowledge, there is no large scale dataset that consistently assessed phenotypic traits concerning metabolic reactions performed by these strains. Therefore, we limited our description to the genotypic traits that differentiate this lineage.

## Conclusions

Comparative genomics and phylogenomic reconstruction allowed the identification of two genera: *Synechococcus and Parasynechococcus*. The two clades and their individual members have marked differences regarding their genetic content, including taxa-specific homologues. This genetic variability pertains to central aspects of the physiology of these organisms and to their interactions with their environment. Future studies should strive to establish how the differences in the genetic content of these taxa affect their lifestyle, specifically with regard to nutritional demands, metabolism, carbon fixation methods and light-utilization strategies.

## List of abbreviations

AAI–Average amino acid identity

ANI–Average nucleotide identity

DDH–DNA-DNA hybridization

HGT–Horizontal gene transfer

MLSA–Multi locus sequence analysis

## Supplemental Information

10.7717/peerj.1555/supp-1 Supplemental Information 1 Orthologous groups. Ortholog composition across the 24 analyzed genomes. Only orthologs present in at least 2 genomes are shown.

10.7717/peerj.1555/supp-2 Supplemental Information 2 Oprhan groups. Orphan composition. List of genes present in only one of each of the 24 genomes with no identified homologs in other genomes.

10.7717/peerj.1522/supp-3 Supplemental Information 3 Phylogenetic reconstruction. Phylogeny of Synechococcus and Prochlorococcus reconstructed based on the concatenated alignments of genes *pyrH, recA* and *gyrB*.

10.7717/peerj.1522/supp-4 Supplemental Information 4 Synteny maps based on whole-genome alignments. (A) Marine *Synechococcus* (B) Freshwater *Synechococcus* (C) Prochlorococcus.

## Additional Information and Declarations

### Data Deposition

The following information was supplied regarding data availability: The research in this article did not generate any raw data.

### New Species Registration

The following information was supplied regarding the registration of a newly described species: Genbank/NCBI

## References

[ref-1] Altschul SF, Gish W, Miller W, Myers EW, Lipman DJ (1990). Basic local alignment search tool. Journal of Molecular Biology.

[ref-2] Batut B, Knibbe C, Marais G, Daubin V (2014). Reductive genome evolution at both ends of the bacterial population size spectrum. Nature Reviews Microbiology.

[ref-3] Bouman HA, Ulloa O, Scanlan DJ, Zwirglmaier K, Li WKW, Platt T, Stuart V, Barlow R, Leth O, Clementson L, Lutz V, Fukasawa M, Watanabe S, Sathyendranath S (2006). Oceanographic basis of the global surface distribution of Prochlorococcus ecotypes. Science.

[ref-4] Chen SL, Hung C-S, Xu J, Reigstad CS, Magrini V, Sabo A, Blasiar D, Bieri T, Meyer RR, Ozersky P, Armstrong JR, Fulton RS, Latreille JP, Spieth J, Hooton TM, Mardis ER, Hultgren SJ, Gordon JI (2006). Identification of genes subject to positive selection in uropathogenic strains of Escherichia coli: a comparative genomics approach. Proceedings of the National Academy of Sciences of the United States of America.

[ref-5] Coenye T, Gevers D, Van de Peer Y, Vandamme P, Swings J (2005). Towards a prokaryotic genomic taxonomy. FEMS Microbiology Reviews.

[ref-6] Colwell RR (1970). Polyphasic taxonomy of the genus vibrio: numerical taxonomy of Vibrio cholerae, Vibrio parahaemolyticus, and related Vibrio species. Journal of Bacteriology.

[ref-7] Darling AE, Mau B, Perna NT (2010). ProgressiveMauve: multiple genome alignment with gene gain, loss and rearrangement. PLoS ONE.

[ref-8] Dufresne A, Ostrowski M, Scanlan DJ, Garczarek L, Mazard S, Palenik BP, Paulsen IT, de Marsac NT, Wincker P, Dossat C, Ferriera S, Johnson J, Post AF, Hess WR, Partensky F (2008a). Unraveling the genomic mosaic of a ubiquitous genus of marine cyanobacteria. Genome Biology.

[ref-9] Dufresne A, Ostrowski M, Scanlan DJ, Garczarek L, Mazard S, Palenik BP, Paulsen IT, de Marsac NT, Wincker P, Dossat C, Ferriera S, Johnson J, Post AF, Hess WR, Partensky F (2008b). Unraveling the genomic mosaic of a ubiquitous genus of marine cyanobacteria. Genome Biology.

[ref-10] Edgar RC (2004). MUSCLE: multiple sequence alignment with high accuracy and high throughput. Nucleic Acids Research.

[ref-11] Fuller NJ, Marie D, Partensky F, Vaulot D, Post AF, Scanlan DJ (2003). Clade-specific 16S ribosomal DNA oligonucleotides reveal the predominance of a single marine Synechococcus clade throughout a stratified water column in the red sea. Applied and Environmental Microbiology.

[ref-12] Glazer AN (1985). Light harvesting by phycobilosomes. Annual Review of Biophysics and Biophysical Chemistry.

[ref-13] Grant JR, Arantes AS, Stothard P (2012). Comparing thousands of circular genomes using the CGView Comparison Tool. BMC Genomics.

[ref-14] Honda D, Yokota A, Sugiyama J (1999). Detection of seven major evolutionary lineages in cyanobacteria based on the 16S rRNA gene sequence analysis with new sequences of five marine Synechococcus strains. Journal of Molecular Evolution.

[ref-15] Iancu CV, Ding HJ, Morris DM, Dias DP, Gonzales AD, Martino A, Jensen GJ (2007). The structure of isolated Synechococcus strain WH8102 carboxysomes as revealed by electron cryotomography. Journal of Molecular Biology.

[ref-16] Juhas M, van der Meer JR, Gaillard M, Harding RM, Hood DW, Crook DW (2009). Genomic islands: tools of bacterial horizontal gene transfer and evolution. FEMS Microbiology Reviews.

[ref-17] Karlin S, Mrazek J, Campbel AM (1997). Compositional biases of bacterial genomes and evolutionary implications. Compositional biases of bacterial genomes and evolutionary implications. Journal of Bacteriology.

[ref-18] Konstantinidis KT, Tiedje JM (2005a). Towards a genome-based taxonomy for prokaryotes. Journal of Bacteriology.

[ref-19] Konstantinidis KT, Tiedje JM (2005b). Genomic insights that advance the species definition for prokaryotes. Proceedings of the National Academy of Sciences of the United States of America.

[ref-20] Kuo C-H, Moran NA, Ochman H (2009). The consequences of genetic drift for bacterial genome complexity. Genome Research.

[ref-21] Laub MT, Goulian M (2007). Specificity in two-component signal transduction pathways. Annual Review of Genetics.

[ref-22] Li WK, Rao DV, Harrison WG, Smith JC, Cullen JJ, Irwin B, Platt T (1983). Autotrophic picoplankton in the tropical ocean. Science.

[ref-23] Li L, Stoeckert CJ, Roos DS (2003). OrthoMCL: identification of ortholog groups for eukaryotic genomes. Genome Research.

[ref-24] Lindell D, Sullivan MB, Johnson ZI, Tolonen AC, Rohwer F, Chisholm SW (2004). Transfer of photosynthesis genes to and from Prochlorococcus viruses. Proceedings of the National Academy of Sciences of the United States of America.

[ref-25] Liu H, Campbell L, Landry MR, Nolla HA, Brown SL, Constantinou J (1998). Prochlorococcus and Synechococcus growth rates and contributions to production in the Arabian Sea during the 1995 Southwest and Northeast monsoons. Deep Sea Research Part II: Topical Studies in Oceanography.

[ref-26] Makarova K, Slesarev A, Wolf Y, Sorokin A, Mirkin B, Koonin E, Pavlov A, Pavlova N, Karamychev V, Polouchine N, Shakhova V, Grigoriev I, Lou Y, Rohksar D, Lucas S, Huang K, Goodstein DM, Hawkins T, Plengvidhya V, Welker D, Hughes J, Goh Y, Benson A, Baldwin K, Lee J-H, Díaz-Muñiz I, Dosti B, Smeianov V, Wechter W, Barabote R, Lorca G, Altermann E, Barrangou R, Ganesan B, Xie Y, Rawsthorne H, Tamir D, Parker C, Breidt F, Broadbent J, Hutkins R, O’Sullivan D, Steele J, Unlu G, Saier M, Klaenhammer T, Richardson P, Kozyavkin S, Weimer B, Mills D (2006). Comparative genomics of the lactic acid bacteria. Proceedings of the National Academy of Sciences of the United States of America.

[ref-27] Mann NH, Cook A, Bailey S, Clokie M, Amanullah A, Azam N, Balliet A, Hollander C, Hoffman BAF, Liebermann D, Zazzeroni F, Papa S, Smaele E De, Franzoso G (2003). Bacterial photosynthesis genes in a virus. Nature.

[ref-28] Matzke NJ, Shih PM, Kerfeld CA (2014). Bayesian analysis of congruence of core genes in Prochlorococcus and Synechococcus and implications on horizontal gene transfer. PLoS ONE.

[ref-29] Meier-Kolthoff JP, Auch AF, Klenk H-P, Göker M (2013). Genome sequence-based species delimitation with confidence intervals and improved distance functions. BMC Bioinformatics.

[ref-30] Moore LR, Ostrowski M, Scanlan DJ, Feren K, Sweetsir T (2005). Ecotypic variation in phosphorus-acquisition mechanisms within marine picocyanobacteria. Aquatic Microbial Ecology.

[ref-31] Mulkidjanian AY, Koonin EV, Makarova KS, Mekhedov SL, Sorokin A, Burd H, Kaznadzey D, Haselkorn R, Wolf YI, Dufresne A, Galperin MY (2006). The cyanobacterial genome core and the origin of photosynthesis. Proceedings of the National Academy of Sciences of the United States of America.

[ref-32] Mullineaux CW (2008). Phycobilisome-reaction centre interaction in cyanobacteria. Photosynthesis Research.

[ref-33] Nakamura Y, Itoh T, Matsuda H, Gojobori T (2004). Biased biological functions of horizontally transferred genes in prokaryotic genomes. Nature Genetics.

[ref-34] Ochman H, Lawrence JG, Groisman EA (2000). Lateral gene transfer and the nature of bacterial innovation. Nature.

[ref-35] Paerl RW, Johnson KS, Welsh RM, Worden AZ, Chavez FP, Zehr JP (2011). Differential distributions of Synechococcus subgroups across the california current system. Frontiers in Microbiology.

[ref-36] Palenik B, Brahamsha B, Larimer FW, Land M, Hauser L, Chain P, Lamerdin J, Regala W, Allen EE, McCarren J, Paulsen I, Dufresne A, Partensky F, Webb EA, Waterbury J (2003). The genome of a motile marine Synechococcus. Nature.

[ref-37] Palenik B, Ren Q, Dupont CL, Myers GS, Heidelberg JF, Badger JH, Madupu R, Nelson WC, Brinkac LM, Dodson RJ, Durkin AS, Daugherty SC, Sullivan SA, Khouri H, Mohamoud Y, Halpin R, Paulsen IT (2006). Genome sequence of Synechococcus CC9311: Insights into adaptation to a coastal environment. Proceedings of the National Academy of Sciences of the United States of America.

[ref-38] Palenik B, Ren Q, Tai V, Paulsen IT (2009). Coastal Synechococcus metagenome reveals major roles for horizontal gene transfer and plasmids in population diversity. Environmental Microbiology.

[ref-39] Partensky F, Blanchot J, Vaulot D (1999). Differential distribution and ecology of Prochlorococcus and Synechococcus in oceanic waters: a review. Bulletin-Institut Oceanographique Monaco-Numero Special.

[ref-40] Partensky F, Hess WR, Vaulot D (1999). Prochlorococcus, a marine photosynthetic prokaryote of global significance. Microbiology and Molecular Biology Reviews.

[ref-41] Post AF, Penno S, Zandbank K, Paytan A, Huse SM, Welch DM (2011). Long term seasonal dynamics of synechococcus population structure in the gulf of aqaba, northern red sea. Frontiers in Microbiology.

[ref-42] Puigbò P, Lobkovsky AE, Kristensen DM, Wolf YI, Koonin EV (2014). Genomes in turmoil: quantification of genome dynamics in prokaryote supergenomes. BMC Biology.

[ref-43] Rae BD, Long BM, Badger MR, Price GD (2013). Functions, compositions, and evolution of the two types of carboxysomes: polyhedral microcompartments that facilitate CO_2_ fixation in cyanobacteria and some proteobacteria. Microbiology and Molecular Biology Reviews.

[ref-44] Richardson TL, Jackson GA (2007). Small phytoplankton and carbon export from the surface ocean. Science.

[ref-45] Robertson BR, Tezuka NR, Watanabe MM (2001). Phylogenetic analyses of Synechococcus strains (cyanobacteria) using sequences of 16S rDNA and part of the phycocyanin operon reveal multiple evolutionary lines and reflect phycobilin content. International Journal of Systematic and Evolutionary Microbiology.

[ref-46] Shih PM, Wu D, Latifi A, Axen SD, Fewer DP, Talla E, Calteau A, Cai F, Tandeau de Marsac N, Rippka R, Herdman M, Sivonen K, Coursin T, Laurent T, Goodwin L, Nolan M, Davenport KW, Han CS, Rubin EM, Eisen JA, Woyke T, Gugger M, Kerfeld CA (2013). Improving the coverage of the cyanobacterial phylum using diversity-driven genome sequencing. Proceedings of the National Academy of Sciences of the United States of America.

[ref-47] Six C, Finkel ZV, Irwin AJ, Campbell DA (2007a). Light variability illuminates niche-partitioning among marine Picocyanobacteria. PLoS ONE.

[ref-48] Six C, Thomas J-C, Garczarek L, Ostrowski M, Dufresne A, Blot N, Scanlan DJ, Partensky F (2007b). Diversity and evolution of phycobilisomes in marine Synechococcus spp.: a comparative genomics study. Genome Biology.

[ref-49] Su Z, Mao F, Dam P, Wu H, Olman V, Paulsen IT, Palenik B, Xu Y (2006). Computational inference and experimental validation of the nitrogen assimilation regulatory network in cyanobacterium Synechococcus sp. WH8102. Nucleic Acids Research.

[ref-50] Sullivan MB, Coleman ML, Weigele P, Rohwer F, Chisholm SW (2005). Three Prochlorococcus cyanophage genomes: signature features and ecological interpretations. PLoS Biology.

[ref-51] Suyama M, Torrents D, Bork P (2006). PAL2NAL: robust conversion of protein sequence alignments into the corresponding codon alignments. Nucleic Acids Research.

[ref-52] Tai V, Palenik B (2009). Temporal variation of Synechococcus clades at a coastal Pacific Ocean monitoring site. The ISME Journal.

[ref-53] Tetu SG, Brahamsha B, Johnson DA, Tai V, Phillippy K, Palenik B, Paulsen IT (2009). Microarray analysis of phosphate regulation in the marine cyanobacterium Synechococcus sp. WH8102. The ISME Journal.

[ref-54] Thompson CC, Vicente AC, Souza RC, Vasconcelos AT, Vesth T, Alves N, Ussery DW, Iida T, Thompson FL (2009). Genomic taxonomy of Vibrios. BMC Evolutionary Biology.

[ref-55] Thompson CC, Silva GG, Vieira NM, Edwards R, Vicente AC, Thompson FL (2013). Genomic taxonomy of the genus prochlorococcus. Microbial Ecology.

[ref-56] Thompson CC, Amaral GR, Campeão M, Edwards RA, Polz MF, Dutilh BE, Ussery DW, Sawabe T, Swings J, Thompson FL (2014). Microbial taxonomy in the post-genomic era: Rebuilding from scratch?. Archives of Microbiology.

[ref-57] Vandamme P, Pot B, Gillis M, Vos P De, Kersters K, Swings J (1996). Polyphasic taxonomy, a consensus approach to bacterial systematics. Microbiological Reviews.

[ref-58] West AH, Stock AM (2001). Histidine kinases and response regulator proteins in two-component signaling systems. Trends in Biochemical Sciences.

[ref-59] Yeates TO, Kerfeld CA, Heinhorst S, Cannon GC, Shively JM (2008). Protein-based organelles in bacteria: carboxysomes and related microcompartments. Nature Reviews Microbiology.

[ref-60] Zhaxybayeva O, Gogarten JP, Charlebois RL, Doolittle WF, Papke RT (2006). Phylogenetic analyses of cyanobacterial genomes: quantification of horizontal gene transfer events. Genome Research.

[ref-61] Zwirglmaier K, Jardillier L, Ostrowski M, Mazard S, Garczarek L, Vaulot D, Not F, Massana R, Ulloa O, Scanlan DJ (2008). Global phylogeography of marine Synechococcus and Prochlorococcus reveals a distinct partitioning of lineages among oceanic biomes. Environmental Microbiology.

