# Supplementary figures and images for: Comparative genomics of Synechococcus and proposal of the new genus Parasynechococcus
Source: PeerJ. 2016 Jan 14;4:e1522. doi: 10.7717/peerj.1522 (PMC4734447; doi:10.7717/peerj.1522)

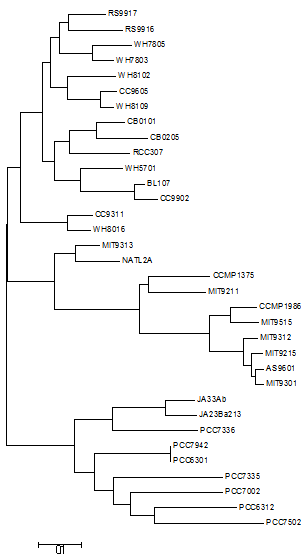

Supplement: Supplemental Information 3 — Phylogeny of Synechococcus and Prochlorococcus reconstructed based on the concatenated alignments of genes pyrH, recA and gyrB. [file peerj-04-1522-s003.png]

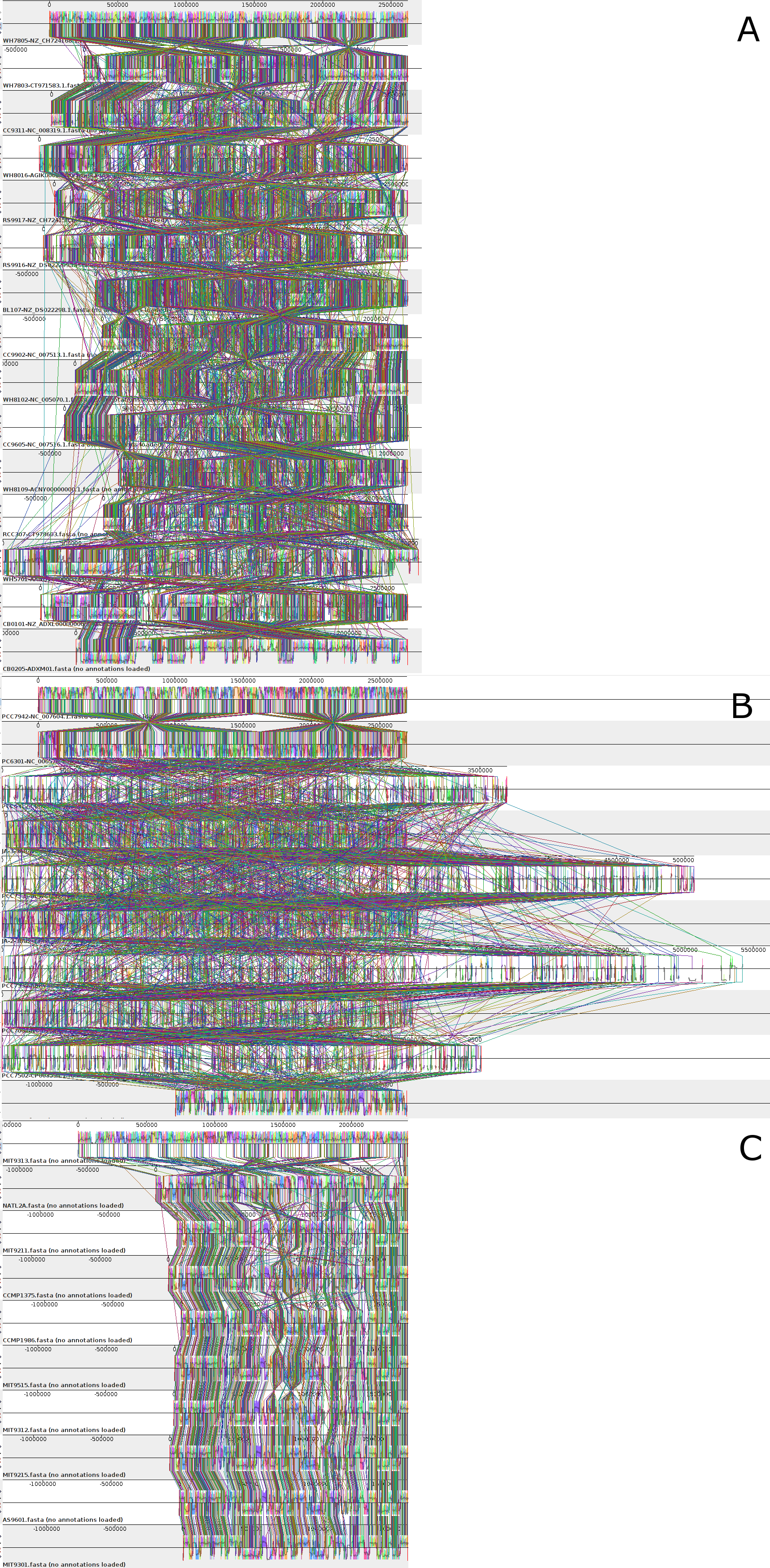

Supplement: Supplemental Information 4 — (A) Marine Synechococcus (B) Freshwater Synechococcus (C) Prochlorococcus. [file peerj-04-1522-s004.png]
